# The Application of Polyrotaxane Cellulose Composite Materials in Quasi-Solid Electrolytes

**DOI:** 10.3390/bioengineering13030292

**Published:** 2026-02-28

**Authors:** Tianyi Wang, Wenzhuo Chen, Yichen Liu, Kailiang Ren, Jin Liang, Jie Kong

**Affiliations:** 1 Shaanxi Key Laboratory of Macromolecular Science and Technology, School of Chemistry and Chemical Engineering, Northwestern Polytechnical University, Xi’an 710072, China; chemwangty@mail.nwpu.edu.cn (T.W.); chenge5232@outlook.com (Y.L.); renkailiang@nwpu.edu.cn (K.R.); jin.liang@nwpu.edu.cn (J.L.); 2 School of Environmental and Chemical Engineering, Xi’an Polytechnic University, Xi’an 710048, China; 20241101@xpu.edu.cn

**Keywords:** cellulose-based composite, gel polymer electrolyte, supramolecular polymer, high-voltage tolerance

## Abstract

Due to its affordability, widespread availability, non-toxicity, biodegradability, and renewability, cellulose is considered a crucial material for addressing the depletion of petroleum resources. In this study, a rotaxane-based supramolecular polymer derived from thermoplastic polyurethane (TPU) was synthesized and combined with cellulose to create a TPU–cellulose composite (TPU-C). This composite was employed as a separator for acrylate-based quasi-solid polymer electrolytes (QPEs). The polymer electrolyte demonstrated a high ionic conductivity of 0.16 mS cm^−1^ at room temperature, a lithium-ion transference number of 0.63, and an electrochemical stability window extending up to 4.7 V. When paired with a LiFePO_4_ (LFP) cathode, the coin cell retained 88.8% of its capacity after 100 cycles at 1 C. A cell assembled with Li and a high-voltage NCM622 cathode maintained a capacity of 65.8% after 100 cycles at 0.3 C. Additionally, the excellent electrochemical performance was analyzed through density functional theory (DFT) calculations to identify the underlying reasons for its outstanding behavior. This study offers new insights into expanding the application potential of cellulose-based composite materials.

## 1. Introduction

In the twenty-first century, the rapid consumption of non-renewable resources such as coal, petroleum, and natural gas, along with other essential commodities, has led to resource depletion and increasingly severe environmental challenges [1,2,3]. As a result, global research efforts have shifted toward innovating and recycling renewable resources and developing environmentally friendly and sustainable materials. These areas have garnered significant attention due to their crucial role in addressing the pressing environmental crises faced by modern society [4,5,6]. Among various renewable materials, cellulose has attracted remarkable interest because of its unique combination of properties, including complete biodegradability, non-toxicity, renewability, ease of modification, chemical stability, and biocompatibility [7,8,9]. With the rapid development of emerging energy technologies, such as smart wearable devices and electric vehicles, the demand for an energy storage system featuring high energy density and enhanced safety has become increasingly critical [10,11].

Lithium metal batteries (LMBs) are regarded as one of the most promising next-generation energy storage systems due to their exceptionally high theoretical specific capacity (3860 mAh g^−1^) and low redox potential (−3.04 V vs. standard hydrogen electrode) [12]. However, commercial liquid-electrolyte lithium batteries face significant safety concerns, such as electrolyte leakage, internal short circuits, and thermal runaway, which can lead to combustion or explosion [13]. To address these issues, replacing conventional liquid electrolytes with solid-state or quasi-solid-state electrolytes has been recognized as an effective strategy to enhance safety and stability, especially in large-scale energy storage applications like electric vehicles [14]. Among various candidates for advanced electrolyte systems, quasi-solid polymer electrolytes (QPEs) have attracted extensive attention due to their unique balance between ionic conductivity and mechanical stability. Unlike liquid electrolytes, which often suffer from leakage and safety risks, or conventional solid polymer electrolytes that generally exhibit limited ion transport capability, QPEs incorporate a controlled amount of liquid plasticizer or solvent into a polymer matrix. This structure enables continuous ion-conducting pathways while maintaining dimensional integrity and suppressing degradation at the electrode–electrolyte interface. QPEs based on supramolecular architectures—such as polyrotaxanes with threaded crown ethers—can provide higher Li^+^ transference numbers and improved interfacial compatibility with electrodes, making them particularly suitable for high-energy-density and safe rechargeable battery systems [15,16,17,18,19]. Nonetheless, they still face challenges such as residual solvent content, limited mechanical robustness, and suboptimal lithium-ion transport, all of which hinder their further development. To overcome these challenges, several strategies have been proposed, including polymer blending, copolymerization, and the incorporation of inorganic fillers or plasticizers. For instance, introducing ion-conducting ceramic fillers into polymer matrices through electrospinning has been shown to enhance both the electrochemical and cycling performance of composite electrolytes. Despite such progress, most polymer matrices currently used in QPEs, such as poly(vinylidene fluoride-hexafluoropropylene) (PVDF-HFP), polyacrylonitrile (PAN), and poly(ethylene oxide) (PEO), still exhibit low lithium-ion transference numbers and insufficient dendrite suppression capabilities, thereby limiting long-term cycling stability [20,21,22]. In this context, cellulose offers a promising bio-based platform for advanced electrolyte design, owing to its natural abundance, renewability, excellent mechanical strength, and tunable surface chemistry. The hierarchical structure and abundant hydroxyl groups of cellulose enable its functionalization or compositing with ion-conducting components, while its robust matrix helps suppress dendrite growth and enhance dimensional stability [23,24,25]. Nevertheless, native cellulose has intrinsic limitations, including insolubility, poor processability, and relatively low ionic conductivity due to the tight packing of its molecular chains. Therefore, modifying cellulose to enhance its ion-conducting properties and mechanical flexibility has become an important strategy for advancing cellulose-based electrolytes. The adoption of high-voltage cathode materials, such as NCM622, significantly enhances the achievable energy density of LMBs [26]. However, the poor compatibility of conventional liquid electrolytes with high-voltage conditions (≥4.2 V) remains a major obstacle to practical application. On the anode side, reactive lithium deposition can degrade polymer electrolytes into parasitic interfacial species—such as Li_2_O, ROCO_2_Li, and Li_2_CO_3_, which have very low ionic conductivity (10^−9^–10^−11^ S cm^−1^) [27,28]. These undesirable reactions lead to uneven lithium plating, dendritic growth, and ultimately poor cycling stability. To address these issues, additives with low LUMO energy levels, such as vinylene carbonate (VC) and lithium nonafluorobutanesulfonate, are often introduced to form sacrificial solid electrolyte interphases (SEI) [27,28]. Another approach involves incorporating lithiophilic lithium-metal alloy species (M = Sn, Sb, Zn, or high-entropy alloys) to lower nucleation barriers and facilitate uniform lithium-ion deposition [29,30]. Nevertheless, forming a stable and flexible SEI layer within intrinsic polymer systems without external fillers remains a considerable challenge for achieving durable lithium-metal interfaces. Covalently bonded polymers and supramolecular polymers based on host–guest recognition are finding increasingly widespread applications in daily life, where host–guest recognition serves as a pivotal strategy for constructing supramolecular polymeric systems [31,32]. Host–guest recognition refers to the process wherein host and guest molecules selectively and reversibly associate via noncovalent interactions, such as hydrogen bonding, van der Waals forces, hydrophobic effects, π–π stacking, and electrostatic interactions. This process plays a crucial role in constructing supramolecular polymers assembled through noncovalent bonds. Among the commonly studied host–guest pairs are cyclodextrins with azobenzene derivatives, calixarenes with pyridinium salts, pillararenes with alkyl cyanides, and crown ethers with amine-based compounds [33,34,35]. Crown ethers, in particular, have attracted sustained research interest due to their straightforward synthesis and strong host–guest recognition capabilities. Crown ether-based polyrotaxanes are a class of supramolecular polymers in which macrocyclic host molecules are threaded onto linear guest chains via a self-assembly process [36,37]. The application of polyrotaxanes in drug delivery, ion separation, and related fields has been increasingly explored [38,39,40,41,42,43,44]. In these structures, the cyclic molecules are mechanically interlocked on the polymer backbone rather than covalently bonded, constrained by bulky end groups. This supramolecular arrangement creates nanochannels formed through noncovalent interactions between the macrocycle and the polymer main chain. Such channels enable selective transport of lithium ions while excluding larger anions [45,46,47]. Unlike α-cyclodextrin, crown ethers consist of oxygen atoms linked by alkyl chains [48]. Owing to their structural similarity to poly(ethylene glycol), crown ethers offer a promising pathway for enhancing the electrochemical performance of quasi-solid polymer electrolytes.

In this study, we report the design and fabrication of a functional cellulose-based composite material that integrates polyrotaxane (PR)-functionalized thermoplastic polyurethane (TPU), achieving dual functionality in both polymer electrolyte membranes. The PR structure comprises 18-crown-6 (18C6) macrocycles threaded onto TPU main chains, forming a dynamic supramolecular network that combines structural flexibility with ion-conductive functionality. Benefiting from spatial confinement effects, the PR framework effectively immobilizes TFSI^−^ anions, leading to a high lithium-ion transference number of 0.63. When employed as a separator in a prototype lithium battery paired with Li, NCM622 and methyl acrylate, the composite exhibited excellent electrochemical stability, maintaining 65.8% capacity retention after 100 cycles at room temperature and stable operation even under a 1.6 C charge–discharge rate. Overall, this study presents a novel strategy for constructing multifunctional cellulose-based materials, successfully integrating supramolecular structure design with electrochemical energy storage functionality within a single system. By combining supramolecular design, polymer electrolyte engineering, and biocompatible functionalization, this approach not only expands the application range of cellulose composites but also provides significant insights for the development of sustainable materials in next-generation energy storage technologies.

## 2. Materials and Methods

### 2.1. General Information of Materials

Polytetrahydrofuran (PTHF, Mn = 2000 Da, 99%), bis(trifluoromethanesulfone)imide (LiTFSI, 99%), 1-methyl-2-pyrrolidinone (NMP, 99%), poly(vinylidene fluoride) (PVDF, 99%), super C65 carbon (99%), dicyclohexylmethane 4,4′-Diisocyanate (DDI, mixture of isomers, 99%), tetrahydrofuran (THF, water ≤ 50 ppm), 1,3-dioxolane (DOL, water ≤ 50 ppm), 1-isocyanato-3,5-bis(trifluoromethyl)benzene (BTFB, 99%), dibutyltin dilaurate (DBTDL, 99%), 1,4,7,10,13,16-hexaoxacyclooctadecane (18C6, 99%), 2,2′-azobis(2-methylpropionitrile) (AIBN, 99%), methyl acrylate, polyethylene glycol diacrylate (PEGDA, 99%) were purchased from Aladdin Biochemical Technology Co., Ltd. (Shanghai, China). All reagents were used without further purification. Lithium metal was purchased from China Energy Lithium Co., Ltd. (Beijing, China) with a thickness of 25 μm. All reactions were carried out under the Schlenk technique.

### 2.2. Synthesis of 2,2′-(1,4-Phenylene)-bis[4-(4-hydroxybutyl)-1,3,2-dioxaborolane (HDB)

**Scheme 1:**
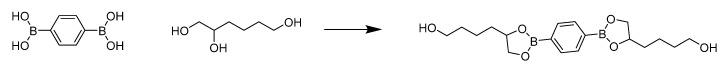
Synthetic route of HDB.

As shown in Scheme 1, the dihydroxyl-bearing boronic ester chain extender (HDB) was synthesized by reacting 1,4-phenylenediboronic acid with 1,2,6-hexanetriol. Typically, 8.3 g (50 mmol) of 1,4-phenylenediboronic acid and 14.8 g (110 mmol) of 1,2,6-hexanetriol were dissolved in 50 mL anhydrous tetrahydrofuran, into which 30.0 g of magnesium sulfate was added. The reaction system was stirred at room temperature for 24 h, followed by filtration and concentration. After solvent removal under reduced pressure, the residue was precipitated in n-hexane to yield the target compound (8.3 g, 90.3%) phenylenediboronic acid with 1,2,6-hexanetriol. The structure is confirmed by the NMR data. ^1^H ^13^C and ^11^B spectra data. ^1^H NMR (CDCl_3_, 500 MHz), δ 7.72 (s, 1H), 4.46 (qd, J = 7.2, 4.9 Hz, 1H), 4.31 (t, J = 8.4 Hz, 1H), 3.83 (dd, J = 9.0, 7.2 Hz, 1H), 3.53 (t, J = 6.3 Hz, 1H), δ 1.63 (dq, J = 13.3, 6.8, 6.0 Hz, 2H), 1.58–1.39 (m, 2H), 1.39–1.29 (m, 2H). ^11^B NMR (CDCl_3_, 160 MHz), δ 26.67. ^13^C NMR (CDCl_3_, 126 MHz), δ 134.01, 77.52, 71.12, 62.27, 35.80, 32.35, 21.33.

### 2.3. Synthesis of Rotaxane Polyurethane (TPU-Rotaxane)

**Scheme 2:**
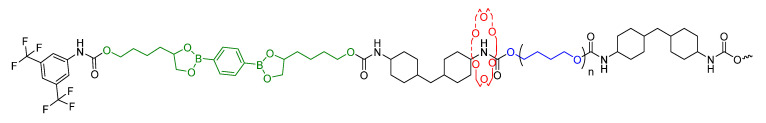
Synthetic route of TPU-Rotaxane.

As shown in Scheme 2, TPU-Rotaxane was synthesized via the condensation of PTHF with DDI. Briefly, 2.5 g (1.25 mmol) of PTHF and 2.5 g (9.46 mmol) of 18C6 were added into a 100 mL Schlenk tube and stirred at 90 °C for 3 h to form a pseudorotaxane. Then cooled to 60 °C, 524 mg (2.00 mmol) of DDI was added, and 5 μL of DBTDL was added to the solution as a reaction catalyst. THF was added to adjust the viscosity of the solution. After stirring for 4 h, HDB 400 mg (0.9 mmol) was added as a chain extender, followed by the addition of 5 μL of DBTDL as a catalyst, and continued stirring for another 4 h. Subsequently, add BTFB as an end-capping agent. After cooling to room temperature, THF was removed by rotary evaporation, and then the sample was placed in an oven at 60 °C for 1 day to remove moisture and trace amounts of THF. Finally, quickly transfer the sample to a glovebox for storage. Before conducting structural characterization, the product was purified by dialyzed against ethanol for 48 h using a dialysis membrane with a molecular weight cut off (MWCO) of 2000 Da. The polymer structure was characterized using ^1^H NMR and NOESY test.

### 2.4. Preparation of the Polyrotaxane-Cellulose Composite Material (TPU-C)

The purified TPU-rotaxane was dissolved in dichloromethane to prepare a concentrated solution with a concentration of 100 mg·mL^−1^. A 2 mL aliquot of this solution was drop-cast onto a circular cellulose film with a thickness of 50 μm. The film was then air-dried to remove the volatile solvent (dichloromethane), followed by placing in a 60 °C oven for 12 h to eliminate any residual solvent, ultimately yielding the TPU-rotaxane cellulose composite material (TPU-C).

### 2.5. Preparation of Electrolyte Precursor Solution

Electrolyte precursor solution was prepared by dissolving 600 mg TPU-rotaxane, 30 mg AIBN, and 670 mg LiTFSI in 2 mL methyl acrylate, vigorously stirred for 4 h at room temperature, to form a homogeneous solution. The salt concentration for the electrolyte was kept constant in all the cases at a molar ratio of 15:1 [-CH_2_CH_2_O-(EO)]/[Li^+^].

### 2.6. Preparation of Cathodes

To prepare the cathode, LFP (Kelude Scientific/Technology Co., Ltd. (Chengdu, China)) and Super C 65 (80/10 wt%) were mixed and ground for 30 min until a uniform powder was obtained. Then, PVDF (10 wt%) was dissolved in NMP, and the mixture was vigorously stirred for 4 h. Finally, the slurry of LiFePO_4_/Super C65/PVDF/NMP was vigorously stirred overnight. The uniform slurry was coated on aluminum foil (mass loading = 1.7 mg cm^−2^) and dried under vacuum at 110 °C for 8 h.

The NCM622 (Kelude Scientific/Technology Co., Ltd. (Chengdu, China)) electrode in the lab was prepared by mixing NCM622, Super C 65, and PVDF dissolved in NMP at a weight ratio of 8:1:1. The slurry was then cast onto aluminum foil and dried at 110 °C under vacuum for 8 h. The average loading mass of the as-prepared NCM811 cathode is approximately 1.7 mg cm^−2^.

### 2.7. Cell Fabrication

CR2016 coin cells Kelude Scientific/Technology Co., Ltd. (Chengdu, China) were assembled in an argon-filled glovebox (≤0.1 ppm H_2_O and ≤0.1 ppm O_2_). Li||NCM622 or Li||LFP cells were assembled by using NCM622 or LFP cathode and lithium foil (50 µm). The charge/discharge measurements were carried out on the NEWARE cycle test system with charge–discharge rates ranging from 0.1 to 1.6 C at 25 °C. 40 μL of polymer precursor solution was dropped onto the TPU-C separator (with a thickness of 50 μm), and then the cell was assembled. The cell was heated at 80 °C for 12 h to fully polymerize the electrolyte.

### 2.8. Analytical Methods

#### 2.8.1. Analysis of Physicochemical Properties

Nuclear magnetic resonance (NMR) was recorded with an Advanced III 400 MHz or Advanced III 500 MHz spectrometer (Bruker, Billerica, MA, USA) at 25 °C. ^1^H NMR, ^13^C NMR or NOESY signals were measured relative to the signal for residual tetramethylsilane (0 ppm) in deuterated chloroform (CDCl_3_), Scanning electron microscope (SEM) measurements were performed on a Verios G4 instrument (Thermo Fisher Scientific, Hillsboro, OR, USA). Linear sweep voltammetry (LSV) and other electrochemical experiments were performed on an electrochemical analyzer/workstation CH Instruments, Inc. (Shanghai, China). The repeated charge and discharge tests were performed on a Neware Technology Limited, Shenzhen, China.

#### 2.8.2. Ionic Conductivity Measurements for Polymer Electrolytes

The electrolyte between two stainless steels assembled in a CR2016 coin cell, 40 μL of polymer precursor solution was dropped onto the TPU-C separator (with a thickness of 50 μm), and then the cell was assembled. The coin cells were assembled and sealed under an argon atmosphere (O_2_ ≤ 0.01 ppm, H_2_O ≤ 0.01 ppm). The cell was heated at 80 °C for 12 h to fully polymerize the electrolyte.

Ionic conductivities (σ) were obtained by an alternating current (AC) impedance measurement to determine the bulk electrolyte resistances ($R_{b}^{0}$) from 20 °C to 60 °C. Impedance spectroscopy measurements were carried out at a frequency range from 1 MHz to 0.1 Hz. The ionic conductivities (σ) were calculated based on Equation (1):

(1) $$\sigma =\frac{L}{R_{b}^{0}\cdot A}$$

#### 2.8.3. Lithium-Ion Transference Number Measurements for Polymer Electrolytes

The electrolyte between two lithium foils was assembled in a CR2016 coin cell, 40 μL of polymer precursor solution was dropped onto the TPU-C separator (with a thickness of 50 μm), and then the cell was assembled. The coin cell was assembled and sealed under an argon atmosphere (O_2_ ≤ 0.01 ppm, H_2_O ≤ 0.01 ppm). The cell was heated at 80 °C for 12 h to fully polymerize the electrolyte.

The Li ion transference number $t_{{Li}^{+}}$ was obtained by the chronoamperometric method and the EIS method. Then, the obtained Li^0^|electrolytes|Li^0^ cells were used to perform a potentiostatic polarization test at an applied voltage of 10 mV. The $t_{{Li}^{+}}$ was calculated from Bruce–Vincent–Evans Equation:

(2) $$t_{{Li}^{+}}=\frac{I_{ss}}{I_{0}}\times \frac{\left(∆V-I_{0}\cdot R_{i}^{0}\right)}{\left(∆V-I_{s}\cdot R_{i}^{ss}\right)}$$

where $I_{0}$ is the initial current, $I_{ss}$ represents the steady state current, $R_{i}^{0}$, $R_{i}^{ss}$ are the initial charge-transfer resistances and steady charge-transfer resistances respectively, ∆V is the applied polarization voltage.

#### 2.8.4. Li Plating-Stripping Measurements for Polymer Electrolytes

Assembly procedure for Li plating-stripping measurements: The electrolytes between two lithium foils were assembled in a 2016 coin cell, 40 μL of polymer precursor solution was dropped onto the TPU-C separator (with a thickness of 50 μm), and then the cell was assembled. The coin cell was assembled and sealed argon atmosphere (O_2_ ≤ 0.01 ppm, H_2_O ≤ 0.01 ppm). The cells were rested at 80 °C for 12 h to fully polymerize the electrolytes. The Li||Li symmetric cells were carried out, stripping and plating test on the NEWARE cycle test system.

#### 2.8.5. SEM Measurements for Li Electrodes in Symmetric Cells

Li electrodes were obtained from symmetric cells and washed with anhydrous THF to remove the residual polymer electrolytes in the glove box under argon. After that, Li electrodes were kept in a flask filled with argon and quickly transferred to the SEM cabin.

#### 2.8.6. Charge–Discharge Cycling Measurements for Polymer Electrolytes

The electrolytes between the lithium foil and cathode were assembled in a 2016 coin cell. 40 μL of polymer precursor solution was dropped onto the TPU-C separator (with a thickness of 50 μm), and then the cell was assembled. The coin cell was assembled and sealed under an argon atmosphere (O_2_ ≤ 0.01 ppm, H_2_O ≤ 0.01 ppm). The cells were rested at 80 °C for 12 h for the full polymerized of electrolytes. The Li|electrolytes|cathode cells were subjected to charge–discharge cycling test on the NEWARE cycle test system.

## 3. Results and Discussion

**Figure 1:**
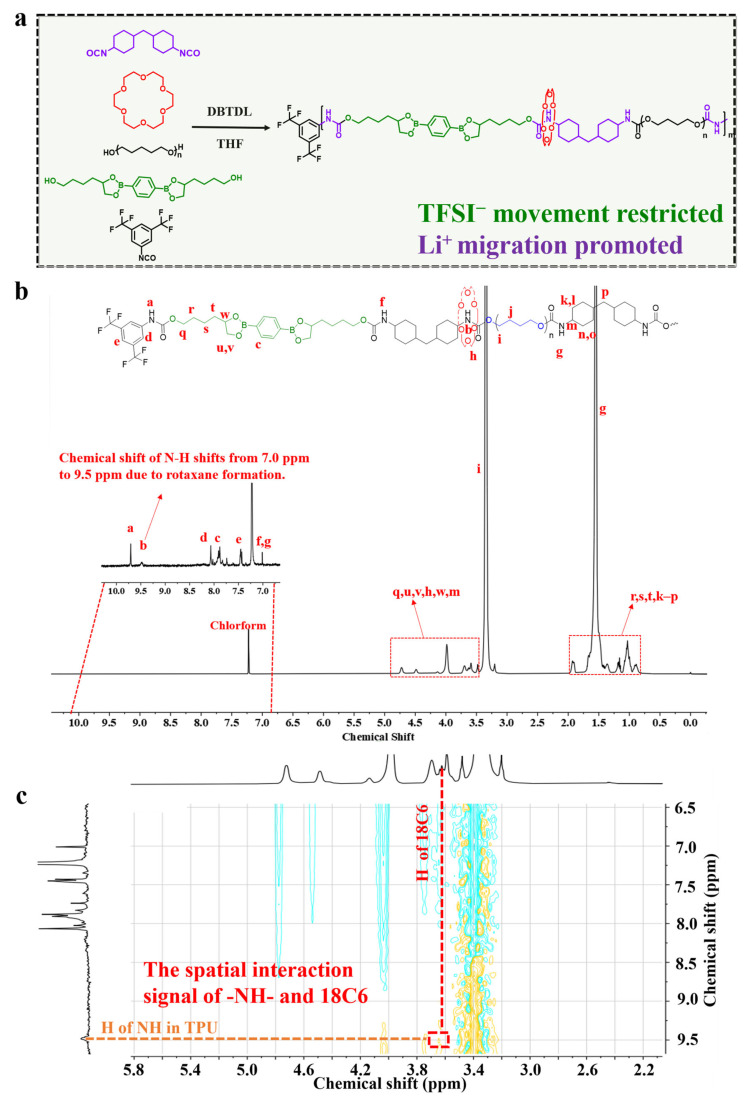
Synthesis and characterization of TPU-rotaxane. (**a**) Design concept of TPU-rotaxane. (**b**) ^1^H NMR spectrum of TPU-rotaxane. (**c**) NOESY spectrum of TPU-rotaxane.

The preparation of cellulose polyrotaxane composite materials involves the synthesis of TPU-rotaxane and the fabrication of TPU-C. The structural design of TPU-rotaxane is shown in Figure 1a. (1) The macrocyclic unit 18C6 threads onto the poly(tetrahydrofuran) (PTHF) chain through hydrogen-bonding interactions, forming a pseudopolyrotaxane. These hydrogen bonds occur between the oxygen atoms of 18C6 and the terminal –OH groups of PTHF. (2) Under the catalysis of dibutyltin dilaurate (DBTDL), the terminal hydroxyl groups of the pseudopolyrotaxane react with isocyanate groups (–N=C=O), which initiated the subsequent polymerization of thermoplastic polyurethane (TPU) polymerization to obtain PR structure. The polymer chains are further extended using boronic ester linkers and end-capped with 1-isocyanato-3,5-bis(trifluoromethyl)benzene (BTFB) to enhance structural stability. The resulting product exhibits excellent solubility and physicochemical stability. As shown in Figure 1a, the ^1^H NMR analysis of TPU-rotaxane reveals the –NH– group (chemical shift at 7.04 ppm) formed by the reaction between isocyanate and PTHF and hydroxyl boronic ester, as well as the –NH– group (chemical shift at 9.74 ppm) formed by the reaction with the end-capping agent BTFB. Additionally, the proton signal of 18C6 (3.66 ppm) and certain –NH– group signals exhibit downfield shifts (from 7.04 ppm to 9.51 ppm), which can be attributed to the deshielding effect induced by hydrogen bonding within the 18C6 ring. Additionally, the NOESY analysis (Figure 1b) reveals a significant spatial correlation between the proton signal of 18C6 and the –NH– group of TPU (indicated by the red dashed rectangle), providing direct evidence for the PR structure.

**Figure 2:**
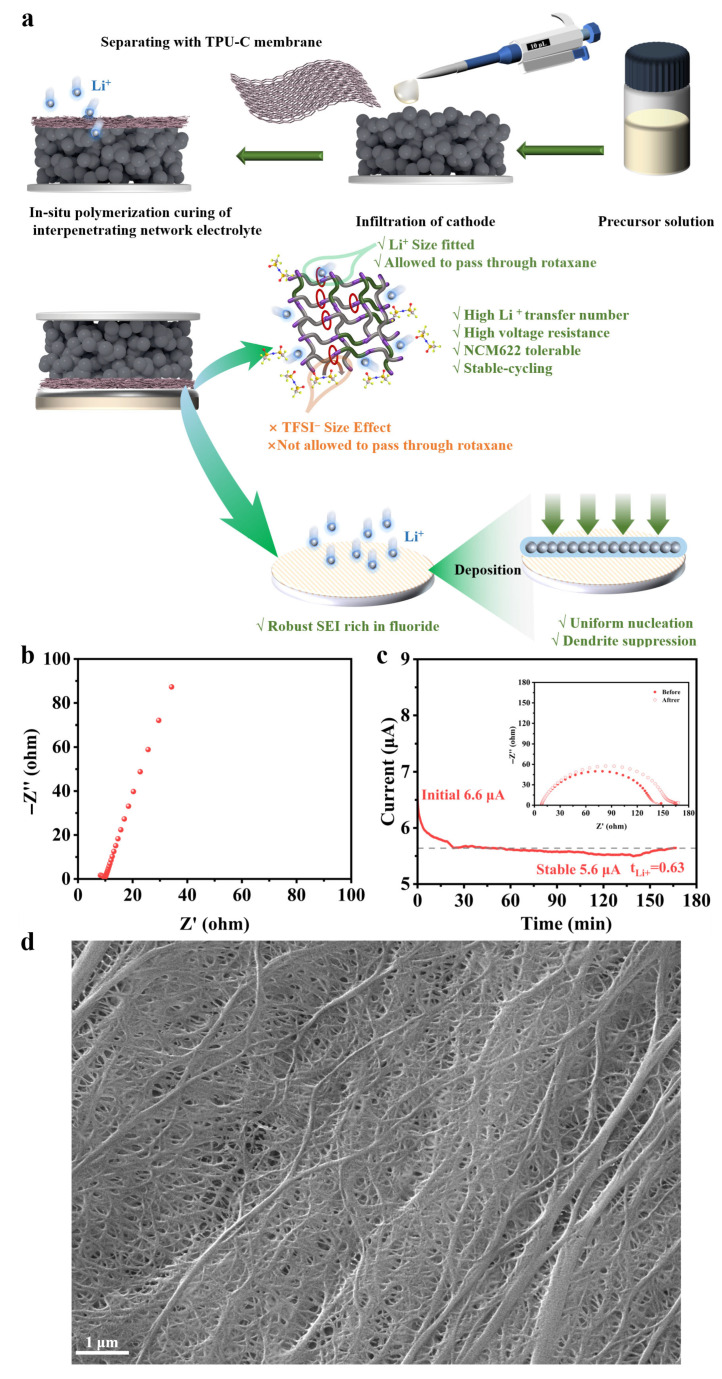
(**a**) Design concept and preparation process of gel electrolyte with TPU-C as the separator. (**b**) Room temperature ionic conductivity of gel electrolyte with TPU-C as the separator. (**c**) Lithium-ion transference number of gel electrolyte with TPU-C as the separator. (**d**) SEM image of TPU-C.

As shown in Figure 2a, the preparation process of the QPE is depicted in Figure 2a. First, LiTFSI, methyl acrylate, the crosslinker polyethylene glycol diacrylate (PEGDA), and the initiator azobisisobutyronitrile (AIBN) are mixed and stirred vigorously to obtain a homogeneously mixed electrolyte precursor solution. This solution is then subjected to in situ polymerization. The resulting solution is assembled with the TPU-C separator, prepared as described above, and placed into a CR2016 battery cell. In situ polymerization is then performed at 80 °C. Finally, the electrolyte is cured in an oven to ensure its uniformity and stability, and is then used for electrochemical performance testing. At 25 °C, the ionic conductivity of the electrolyte reaches 0.16 mS cm^−1^, indicating accelerated Li^+^ diffusion kinetics. Figure 2b shows that the electrolyte with TPU-C separator exhibits a high lithium-ion transference number ($t_{{Li}^{+}}$) = 0.63. This improvement is attributed to the restricted migration of TFSI^−^ anions in the electrolyte. Due to the significantly larger radius of the TFSI^−^ anion (3.30 Å) compared to the cavity diameter of the 18C6 host molecule (1.60 Å), the rotaxane structure spatially confines the TFSI^−^ anion, preventing its entry and movement. In contrast, the smaller Li^+^ (radius 0.76 Å) can selectively pass through this structure [49,50,51]. Additionally, the Lewis acid sites in the SSNE further adsorb the TFSI^−^ anion, which further restricts its migration, thereby enhancing the lithium-ion transference number. As shown in Figure 2d, the SEM image of TPU-C reveals a uniform morphology with no apparent phase separation after incorporating TPU-rotaxane and cellulose into the composite. This indicates good compatibility between the TPU-rotaxane and the cellulose matrix, further confirming the successful fabrication of the rotaxane-cellulose composite material.

**Figure 3:**
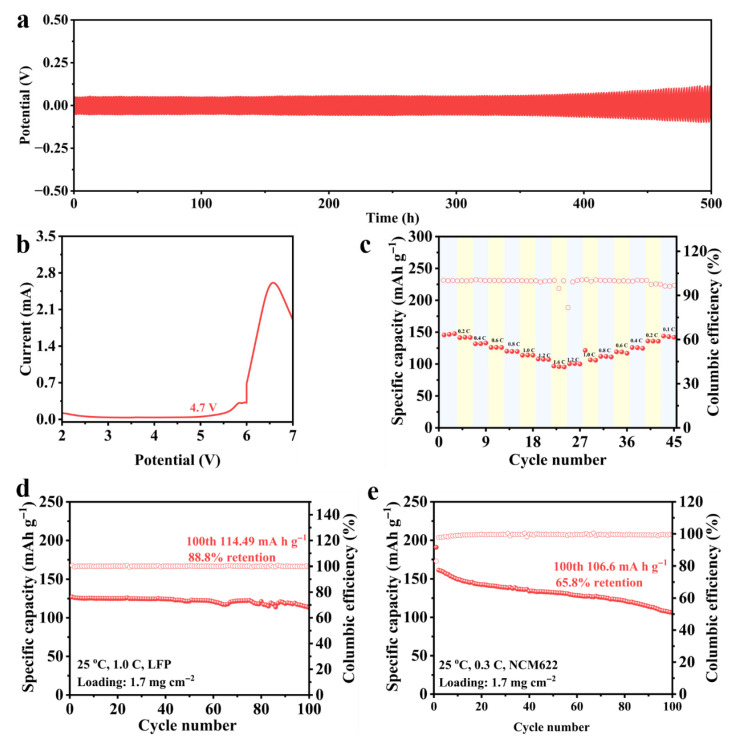
Electrochemical performance of gel electrolyte with TPU-C as the separator. (**a**) Lithium deposition and stripping performance. (**b**) LSV curve of TPU-C QPE. (**c**) Rate performance of Li|TPU-C QPE|LFP cell (Solid circles denote the discharge specific capacity; hollow circles denote the Coulombic efficiency). (**d**) Cycling performance of Li|TPU-C QPE |LFP cell at 1 C (Solid circles denote the discharge specific capacity; hollow circles denote the Coulombic efficiency). (**e**) Cycling performance of Li|TPU-C QPE |NCM622 cell at 0.3 C (Solid circles denote the discharge specific capacity; hollow circles denote the Coulombic efficiency).

Upon analysis of Figure 3a, the Li||Li symmetrical cell assembled with TPU-C as the separator exhibits excellent cycling stability at room temperature. The cell operates stably for over 600 h at a current density of 0.2 mA cm^−2^, with a polarization voltage of approximately 35 mV, demonstrating the separator’s excellent dendrite suppression capability. After 200 h of cycling, the lithium symmetrical cell was disassembled, and its surface was analyzed by SEM. As shown in Figure S1, no dendrite formation was observed on the lithium surface after 200 h of cycling, which confirms that the QPE with TPU-C separator has outstanding lithium dendrite suppression ability. The LSV curve was then tested (Figure 3b), showing that the electrochemical stability window of the system with TPU-C as the separator reaches up to 4.7 V. This indicates that the system has excellent high-voltage resistance performance. Benefiting from the high-voltage tolerance of the electrolyte and its favorable interfacial kinetics with lithium metal foil, the LFP||Li cell using TPU-C separator exhibits excellent rate performance (Figure 3c), achieving stable capacity output under various charge–discharge rates. Under a cutoff voltage of 4.0 V, the battery delivers discharge capacities of 141.4 mAh g^−1^, 113.8 mAh g^−1^, and 96.8 mAh g^−1^ at 0.2 C, 1.0 C, and 1.6 C rates, respectively. When the rate is returned to 0.2 C, the specific capacity recovers to 136.1 mAh g^−1^, confirming that the electrolyte employing the TPU-C separator possesses excellent structural and interfacial stability. At room temperature and a rate of 1.0 C, the cell with a TPU-C separator retains 88.8% of its initial capacity after 100 cycles. Even when paired with the high-voltage cathode material NCM622, the battery maintains 65.8% of its initial capacity after 100 cycles at 0.3 and a cutoff voltage of 4.3 V. These results clearly demonstrate the excellent cycling stability and high-voltage tolerance of the TPU-rotaxane composite separator–based electrolyte system.

**Figure 4:**
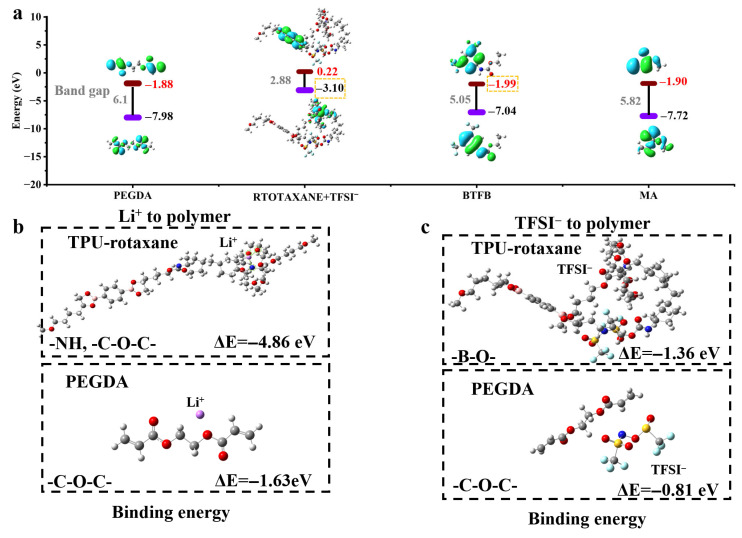
The orbital energy levels of each component in the QPEs system and the binding energy of ions with TPU-ROTAXANE and PEGDA. (**a**) The HOMO-LUMO energy levels of each component in the QPE. (**b**) The binding energy of TPU-ROTAXANE and PEGDA with Li^+^, (**c**) The binding energy of TPU-ROTAXANE and PEGDA with TFSI^−^.

Based on the excellent performance of the electrolyte mentioned earlier, we conducted a theoretical analysis to investigate the reasons for its outstanding performance. The electrochemical stability of SSNE was systematically evaluated by analyzing the electrolyte components. Through density functional theory (DFT) calculations, the molecular orbital energy levels of TPU-ROTAXANE and TFSI^−^ complex (ROTAXANE&TFSI^−^), PEGDA, end-capping agent (BTFB), and methyl acrylate (MA) were determined (Figure 4a). The highest occupied molecular orbital (HOMO) and lowest unoccupied molecular orbital (LUMO) energy levels are important indicators for describing redox behavior. Components with the highest HOMO are preferentially oxidized at the cathode, forming a stable cathode electrolyte interphase (CEI) that inhibits polymer degradation and metal dissolution. Furthermore, components with the lowest LUMO are preferentially reduced at the anode, forming a uniform solid electrolyte interphase (SEI) that promotes Li^+^ diffusion, suppresses dendrite formation, and enhances cycling stability.

The DFT results indicate that the HOMO energy levels of PEGDA, ROTAXANE&TFSI^−^, BTFB, TTE, and MA are −7.98, −3.10, −7.04, and −7.72 eV, respectively. These values suggest that in GPE, ROTAXANE&TFSI^−^ is preferentially oxidized to form a stable CEI. The LUMO energy levels are −1.88, 0.22, −1.99, and 1.90 eV, indicating that in GPEs, BTFB is preferentially reduced to form a stable SEI. This suggests that TPU-C not only forms a stable CEI at the positive electrode to inhibit the decomposition of GPEs, but also its components generate a stable SEI at the negative electrode, suppressing lithium dendrite growth. These combined factors contribute to the excellent electrochemical performance of GPEs.

According to the DFT calculations (Figure 4b,c), the binding energy of Li^+^ with TPU-rotaxane (−4.86 eV) is significantly stronger than its binding energy with PEGDA (−1.63 eV). Additionally, the adsorption energy of TFSI^−^ on the -O-B-O- group of TPU-rotaxane (−1.36 eV) is much higher than its adsorption energy on the C-O-C group of PDOL (−0.81 eV). These results further confirm that the migration of TFSI^−^ in the GPE is limited, thereby facilitating Li^+^ transport, significantly improving the lithium-ion transference number, reducing concentration polarization at the lithium anode, suppressing dendrite growth, and enhancing the electrochemical performance of GPE. As presented in Table 1, the gel electrolyte system exhibits excellent comprehensive performance, which further demonstrates the promising application prospects of introducing a supramolecular polymer system into polymer electrolytes [52,53,54,55].

## 4. Conclusions

In this study, a novel composite separator based on the supramolecular polyrotaxane TPU-rotaxane and cellulose was successfully designed and fabricated, and applied in quasi-solid-state lithium-ion batteries. The developed TPU-C composite electrolyte demonstrated outstanding overall performance: it achieved a room-temperature ionic conductivity of 0.16 mS·cm^−1^, a high lithium-ion transference number of 0.63, and a wide electrochemical stability window up to 4.7 V (vs. Li^+^/Li). These properties enabled compatibility with high-voltage cathode materials such as NCM622. The corresponding cell retained 65.8% of its capacity after 100 cycles at 0.3 C, indicating good chemical and electrochemical stability at the electrolyte/electrode interface and effective suppression of interfacial side reactions under high-voltage conditions. Mechanistic studies revealed that the unique dynamic sliding behavior of the polyrotaxane component facilitates the construction of continuous ion transport pathways, promotes directional migration of lithium ions, and mitigates concentration polarization during cycling, thereby laying the foundation for high-rate performance. The progress achieved by this material system in key performance metrics offers an innovative strategy to overcome the long-standing challenge of simultaneously enhancing ionic conductivity and interfacial stability in polymer electrolytes.

## Figures and Tables

**Table 1:** The comparison of cell parameters of Li cells.

Temperature (°C)	Transference Number	Capacity Retention (Cycle Number)	Cathode	Ref.
25	0.63	88.8% (100)	LFP	This work
25	0.63	65.8% (100)	NCM622	This work
25	/	82.4% (300)	NCM811	[52]
25	0.68	85.2% (120)	LFP	[53]
30	0.72	84.3 (100)	NCM811	[54]
30	0.58	99.5% (250)	C/S	[55]

## Data Availability

The original contributions presented in this study are included in the article/Supplementary Materials. Further inquiries can be directed to the corresponding author.

## Supplementary Materials

The following supporting information can be downloaded at: https://www.mdpi.com/article/10.3390/bioengineering13030292/s1, Figure S1: SEM image of cycled Li anode. Figure S2: ^1^H NMR spectrum of HDB. Figure S3: ^11^B NMR spectrum of HDB. Figure S4: ^13^C NMR spectrum of HDB. References [56,57].
