# Supplementary material for: The Application of Polyrotaxane Cellulose Composite Materials in Quasi-Solid Electrolytes
Source: Bioengineering (Basel). 2026 Feb 28;13(3):292. doi: 10.3390/bioengineering13030292 (PMC13023748; doi:10.3390/bioengineering13030292)
Supplement: Supplementary file 1 [file bioengineering-13-00292-s001.zip › bioengineering-4107303-supplementary.pdf]

Supporting Information

The Application of Polyrotaxane Cellulose Composite Materials in Quasi-Solid Electrolytes

Tianyi Wang [1,†], Wenzhuo Chen [2,†], Yichen Liu [1], Kailiang Ren [1], Jin Liang [1] and Jie Kong [1,]*

[1]Shaanxi Key Laboratory of Macromolecular Science and Technology, School of Chemistry and Chemical Engineering, Northwestern Polytechnical University, Xi'an 710072, China

[2]School of Environmental and Chemical Engineering, Xi'an Polytechnic University, Xi'an 710048, China

*Corresponding Authors, E-mail: kongjie@nwpu.edu.cn (J.K.)

[†]These authors contributed equally to this work.

1. Computational Details

The theoretical calculations were performed using the Gaussian 16 suite of programs.[S1] All the molecules were optimized at the B3LYP/6-31+g(d) level of theory. The structures were characterized as a local energy minimum on the potential energy surface. The molecular orbital levels of the above-mentioned molecules were studied via theoretical calculations, including the highest occupied molecular orbital (HOMO) and the lowest unoccupied molecular orbital (LUMO). In addition, the color-filled iso-surface graphs of molecules were employed to visualize molecular orbitals with the aid of a visual molecular dynamics program.[S2] The calculations of bending energy are based on the Gaussian 16 suite of programs. The optimization of molecular geometries and subsequent single-point energy calculations were performed at the B3LYP/6-31+g(d) and B3LYP/6-31+g(d, p) levels, respectively. The binding energies (E_b) were calculated as follows.

$$E_b = E_{complex} - \sum E_{fragment} \quad \text{S1}$$

2. Characterization data

2.1 SEM image of anode

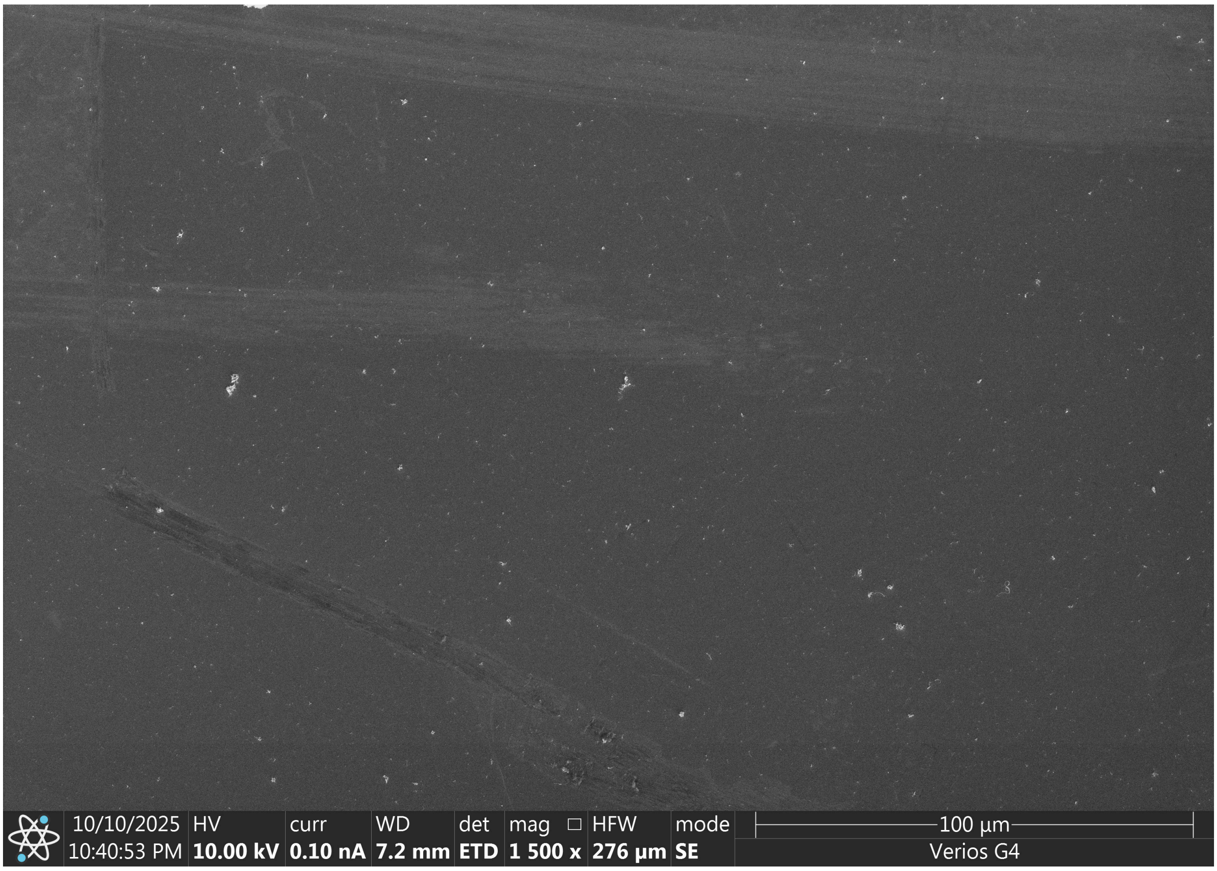

Figure S1. SEM image of cycled Li anode.

3.2 ^{1}H, ^{11}B and ^{13}C NMR spectra of HDB

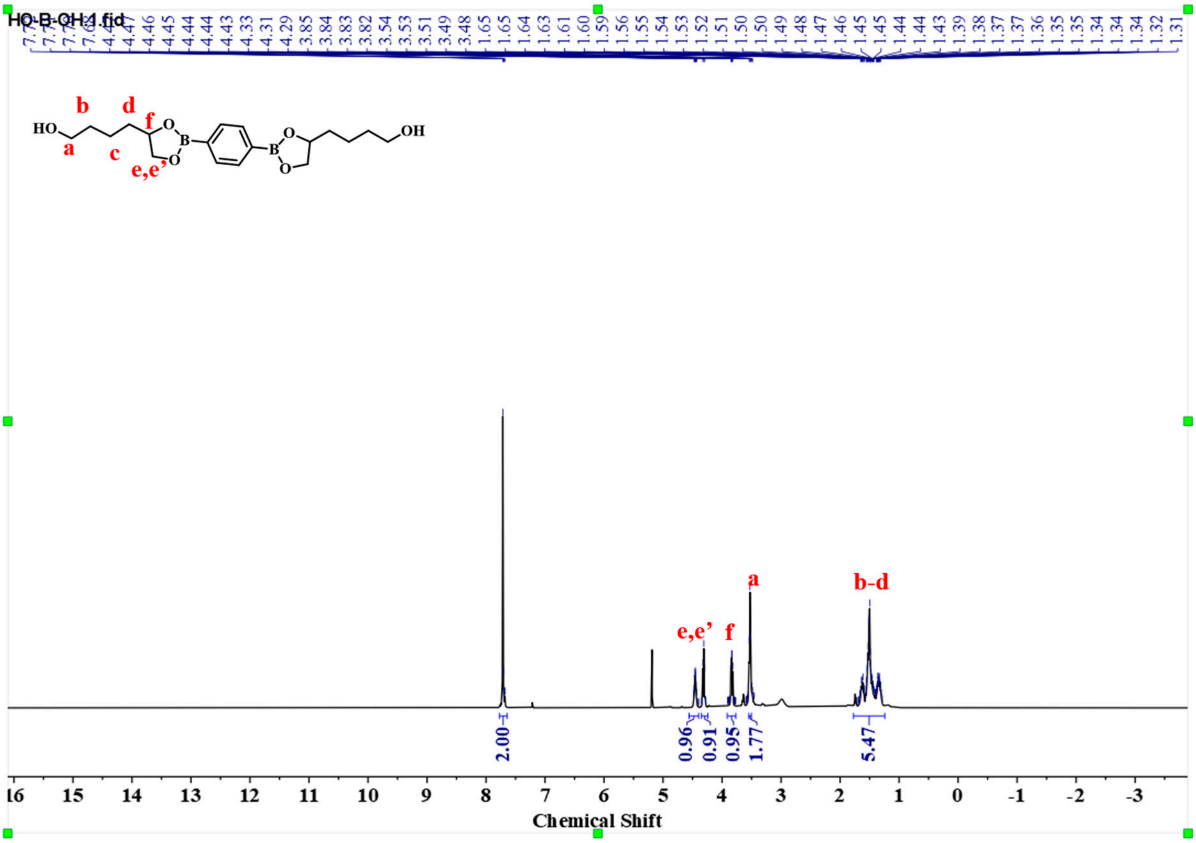

Figure S2. ^{1}H NMR spectrum of HDB.

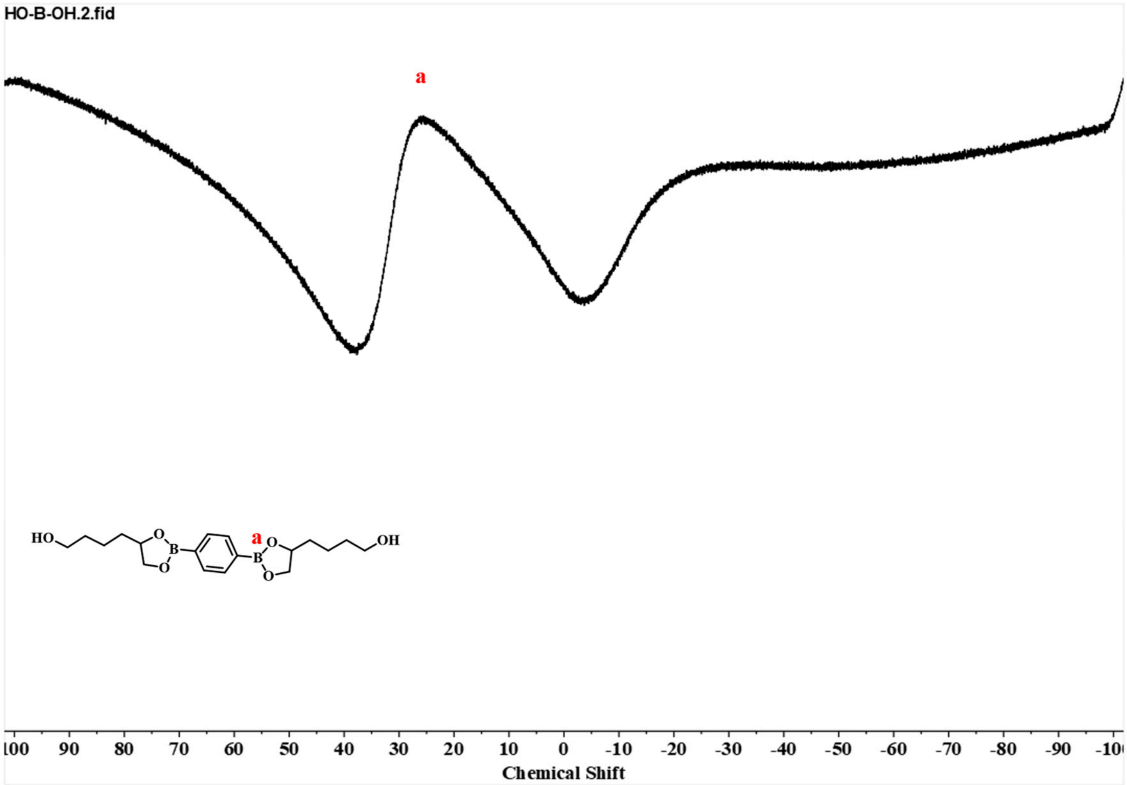

Figure S3. ^{11}B NMR spectrum of HDB.

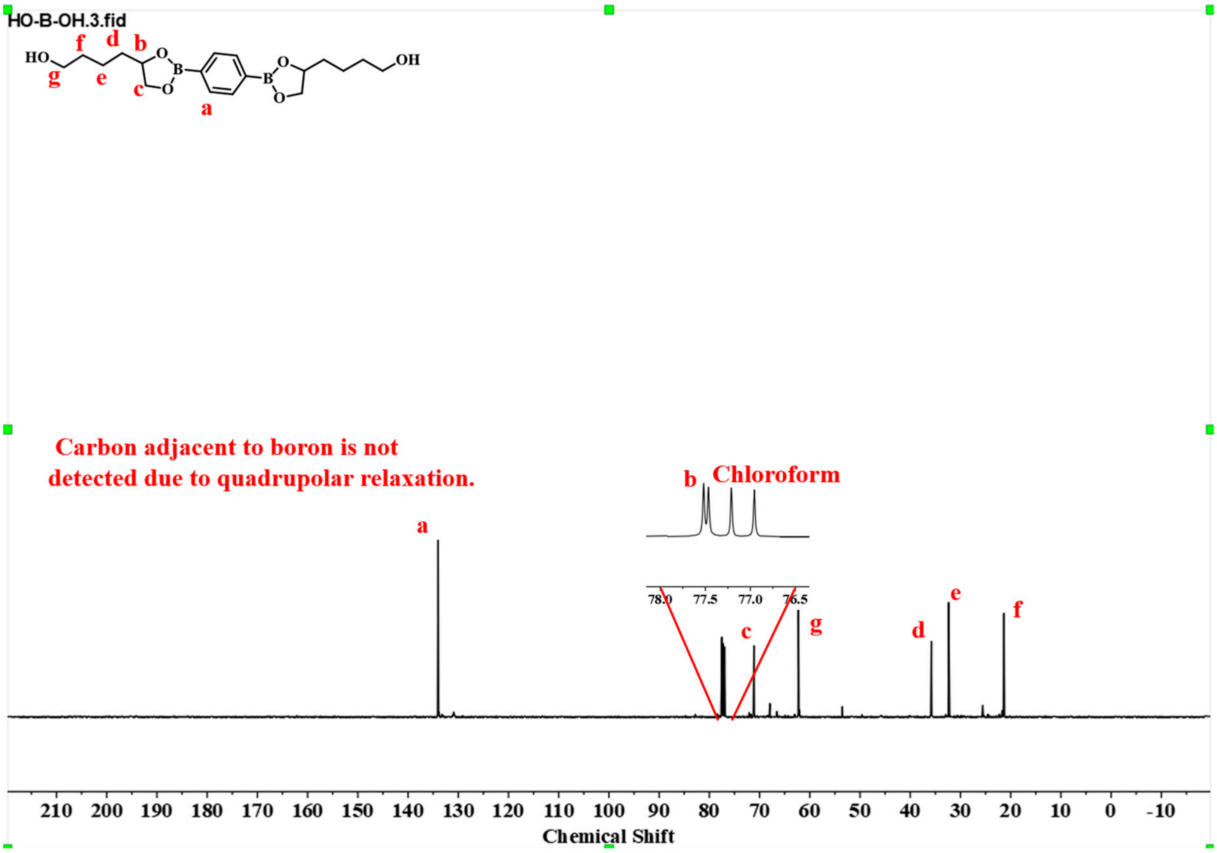

Figure S4. ^{13}C NMR spectrum of HDB.

[S1] M. Frisch, G. Trucks, H. Schlegel, G. Scuseria, M. Robb, J. Cheeseman, G. Scalmani, V. Barone, G. Petersson, H. Nakatsuji, *Gaussian16 (Revision A. 03),* **2016**.

[S2] W. Humphrey, A. Dalke, K. Schulten, *J. Mol. Graphics* **1996**, *14*, 33.
